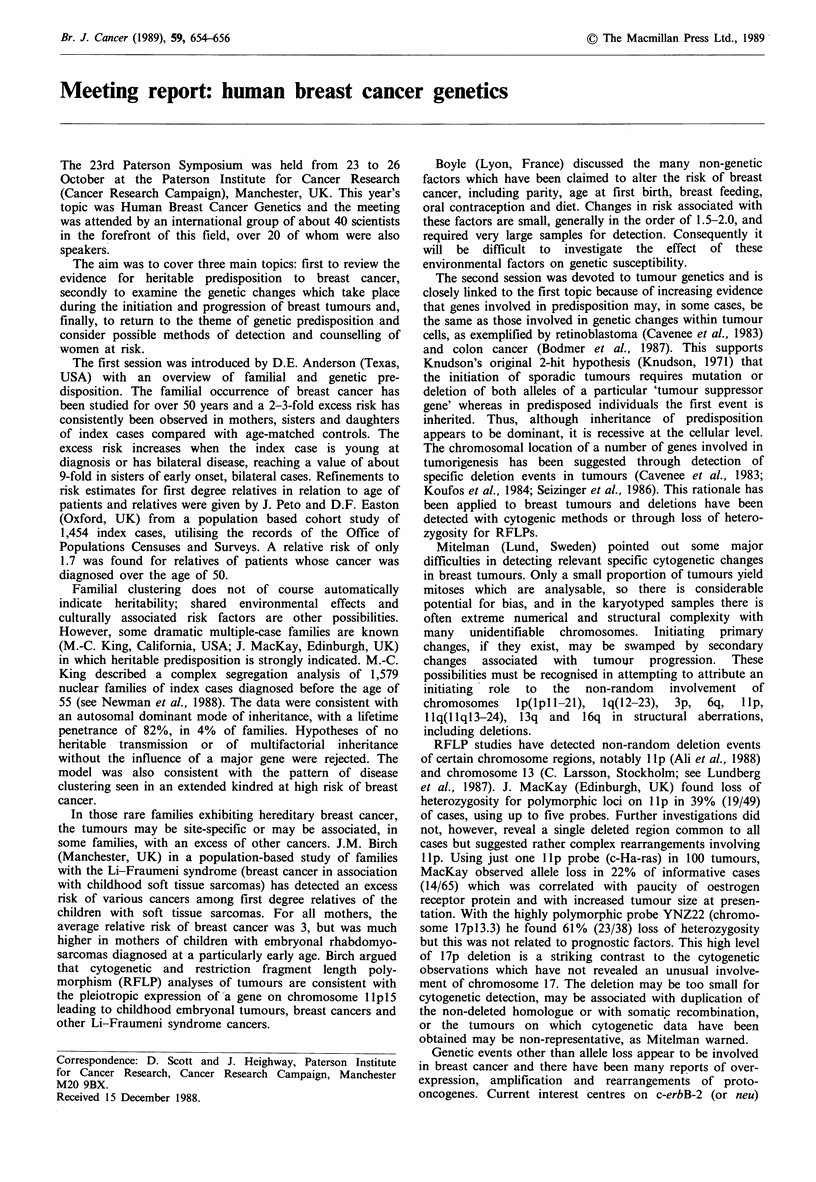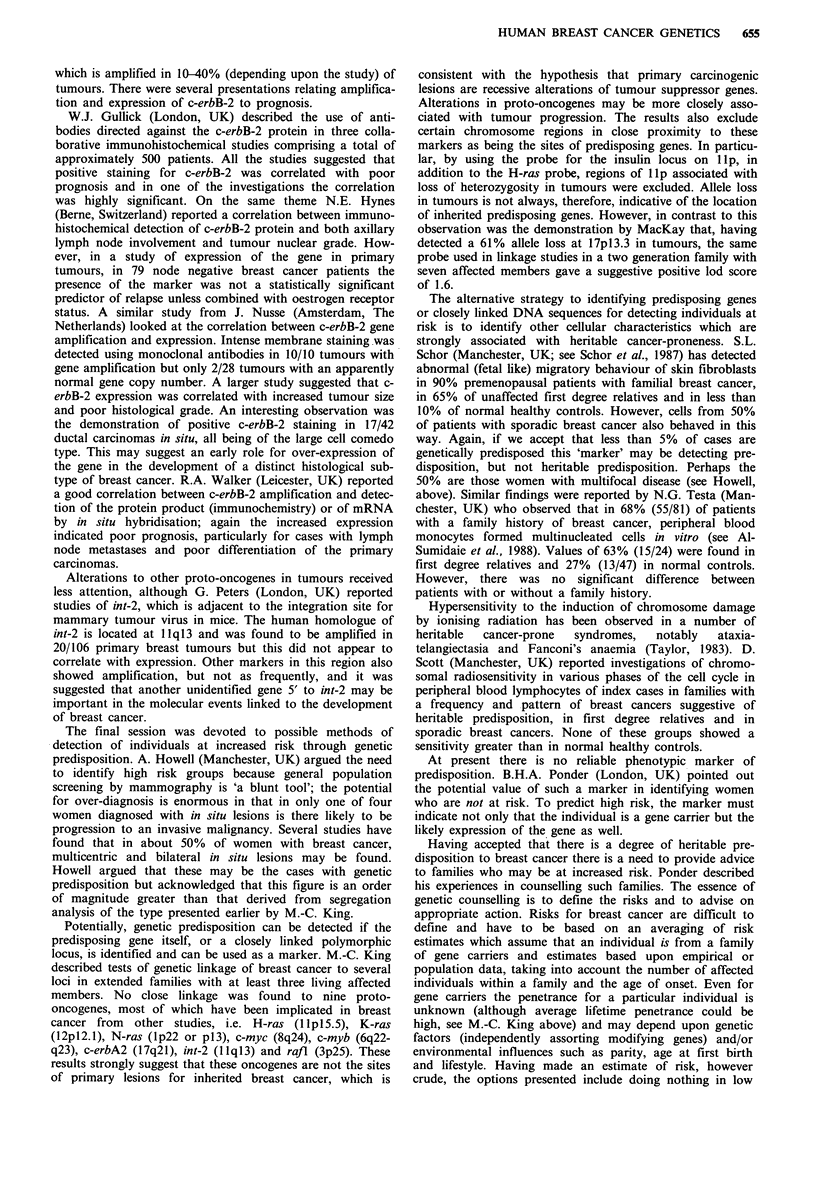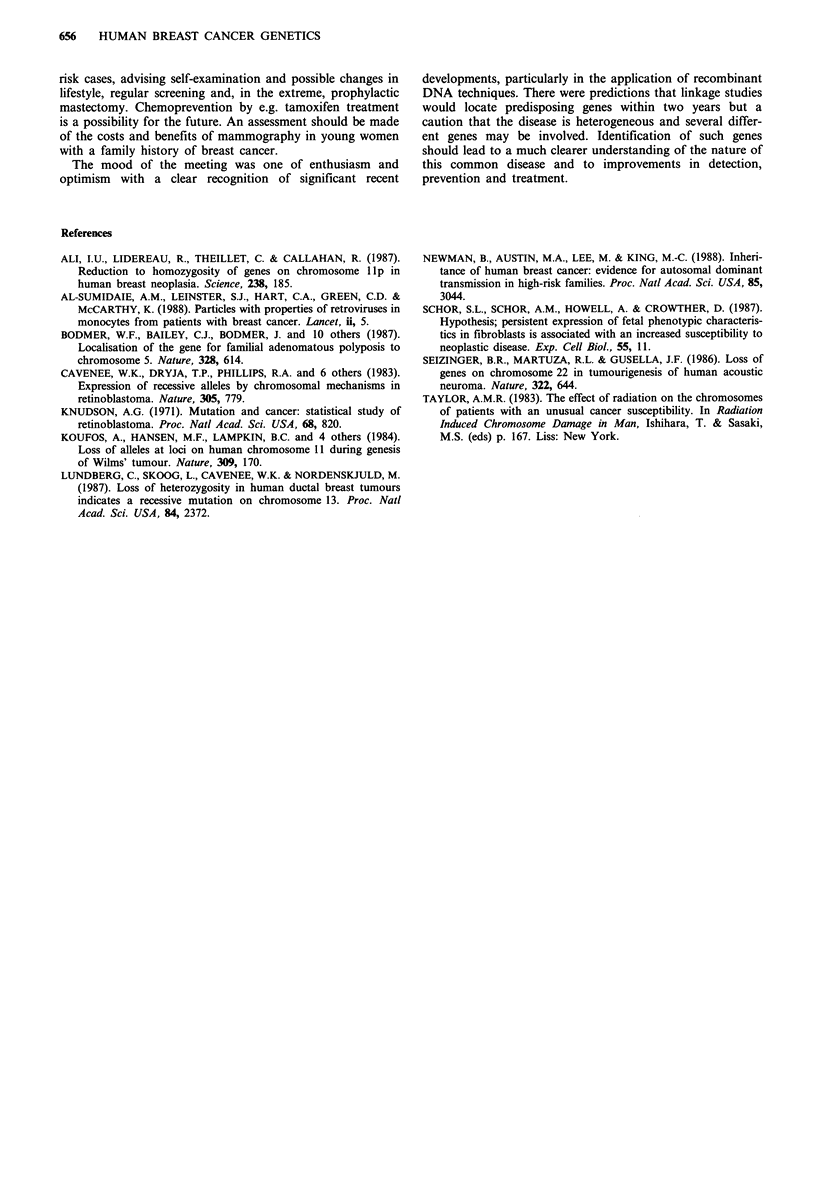# 23rd Paterson Symposium: human breast cancer genetics - 23-26 October, 1988

**Published:** 1989-04

**Authors:** 


*Br. J. Cancer* (1989), **59**, 654–656 © The Macmillan Press Ltd., 1989

### Meeting report: human breast cancer genetics

The 23rd Paterson Symposium was held from 23 to 26 October at the Paterson Institute for Cancer Research (Cancer Research Campaign), Manchester, UK. This year's topic was Human Breast Cancer Genetics and the meeting was attended by an international group of about 40 scientists in the forefront of this field, over 20 of whom were also speakers.

The aim was to cover three main topics: first to review the evidence for heritable predisposition to breast cancer, secondly to examine the genetic changes which take place during the initiation and progression of breast tumours and, finally, to return to the theme of genetic predisposition and consider possible methods of detection and counselling of women at risk.

The first session was introduced by D.E. Anderson (Texas, USA) with an overview of familial and genetic predisposition. The familial occurrence of breast cancer has been studied for over 50 years and a 2–3-fold excess risk has consistently been observed in mothers, sisters and daughters of index cases compared with age-matched controls. The excess risk increases when the index case is young at diagnosis or has bilateral disease, reaching a value of about 9-fold in sisters of early onset, bilateral cases. Refinements to risk estimates for first degree relatives in relation to age of patients and relatives were given by J. Peto and D.F. Easton (Oxford, UK) from a population based cohort study of 1,454 index cases, utilising the records of the Office of Populations Censuses and Surveys. A relative risk of only 1.7 was found for relatives of patients whose cancer was diagnosed over the age of 50.

Familial clustering does not of course automatically indicate heritability; shared environmental effects and culturally associated risk factors are other possibilities. However, some dramatic multiple-case families are known (M.-C. King, California, USA; J. MacKay, Edinburgh, UK) in which heritable predisposition is strongly indicated. M.-C. King described a complex segregation analysis of 1,579 nuclear families of index cases diagnosed before the age of 55 (see Newman *et al.*, 1988). The data were consistent with an autosomal dominant mode of inheritance, with a lifetime penetrance of 82%, in 4% of families. Hypotheses of no heritable transmission or of multifactorial inheritance without the influence of a major gene were rejected. The model was also consistent with the pattern of disease clustering seen in an extended kindred at high risk of breast cancer.

In those rare families exhibiting hereditary breast cancer, the tumours may be site-specific or may be associated, in some families, with an excess of other cancers. J.M. Birch (Manchester, UK) in a population-based study of families with the Li–Fraumeni syndrome (breast cancer in association with childhood soft tissue sarcomas) has detected an excess risk of various cancers among first degree relatives of the children with soft tissue sarcomas. For all mothers, the average relative risk of breast cancer was 3, but was much higher in mothers of children with embryonal rhabdomyosarcomas diagnosed at a particularly early age. Birch argued that cytogenetic and restriction fragment length polymorphism (RFLP) analyses of tumours are consistent with the pleiotropic expression of a gene on chromosome 11p15 leading to childhood embryonal tumours, breast cancers and other Li–Fraumeni syndrome cancers.

Boyle (Lyon, France) discussed the many non-genetic factors which have been claimed to alter the risk of breast cancer, including parity, age at first birth, breast feeding, oral contraception and diet. Changes in risk associated with these factors are small, generally in the order of 1.5–2.0, and required very large samples for detection. Consequently it will be difficult to investigate the effect of these environmental factors on genetic susceptibility.

The second session was devoted to tumour genetics and is closely linked to the first topic because of increasing evidence that genes involved in predisposition may, in some cases, be the same as those involved in genetic changes within tumour cells, as exemplified by retinoblastoma (Cavenee *et al.*, 1983) and colon cancer (Bodmer *et al.*, 1987). This supports Knudson's original 2-hit hypothesis (Knudson, 1971) that the initiation of sporadic tumours requires mutation or deletion of both alleles of a particular 'tumour suppressor gene' whereas in predisposed individuals the first event is inherited. Thus, although inheritance of predisposition appears to be dominant, it is recessive at the cellular level. The chromosomal location of a number of genes involved in tumorigenesis has been suggested through detection of specific deletion events in tumours (Cavenee *et al.*, 1983; Koufos *et al.*, 1984; Seizinger *et al.*, 1986). This rationale has been applied to breast tumours and deletions have been detected with cytogenic methods or through loss of heterozygosity for RFLPs.

Mitelman (Lund, Sweden) pointed out some major difficulties in detecting relevant specific cytogenetic changes in breast tumours. Only a small proportion of tumours yield mitoses which are analysable, so there is considerable potential for bias, and in the karyotyped samples there is often extreme numerical and structural complexity with many unidentifiable chromosomes. Initiating primary changes, if they exist, may be swamped by secondary changes associated with tumour progression. These possibilities must be recognised in attempting to attribute an initiating role to the non-random involvement of chromosomes 1p(1p11–21), 1q(12–23), 3p, 6q, 11p, 11q(11q13–24), 13q and 16q in structural aberrations, including deletions.

RFLP studies have detected non-random deletion events of certain chromosome regions, notably 11p (Ali *et al.*, 1988) and chromosome 13 (C. Larsson, Stockholm; see Lundberg *et al.*, 1987). J. MacKay (Edinburgh, UK) found loss of heterozygosity for polymorphic loci on 11p in 39% (19/49) of cases, using up to five probes. Further investigations did not, however, reveal a single deleted region common to all cases but suggested rather complex rearrangements involving 11p. Using just one 11p probe (c-Ha-ras) in 100 tumours, MacKay observed allele loss in 22% of informative cases (14/65) which was correlated with paucity of oestrogen receptor protein and with increased tumour size at presentation. With the highly polymorphic probe YNZ22 (chromosome 17p13.3) he found 61% (23/38) loss of heterozygosity but this was not related to prognostic factors. This high level of 17p deletion is a striking contrast to the cytogenetic observations which have not revealed an unusual involvement of chromosome 17. The deletion may be too small for cytogenetic detection, may be associated with duplication of the non-deleted homologue or with somatic recombination, or the tumours on which cytogenetic data have been obtained may be non-representative, as Mitelman warned.

Genetic events other than allele loss appear to be involved in breast cancer and there have been many reports of overexpression, amplification and rearrangements of proto-oncogenes. Current interest centres on c-*erb*B-2 (or *neu*)

Correspondence: D. Scott and J. Heighway, Paterson Institute for Cancer Research, Cancer Research Campaign, Manchester M20 9BX.
Received 15 December 1988.

which is amplified in 10–40% (depending upon the study) of tumours. There were several presentations relating amplification and expression of c-*erb*B-2 to prognosis.

W.J. Gullick (London, UK) described the use of antibodies directed against the c-*erb*B-2 protein in three collaborative immunohistochemical studies comprising a total of approximately 500 patients. All the studies suggested that positive staining for c-*erb*B-2 was correlated with poor prognosis and in one of the investigations the correlation was highly significant. On the same theme N.E. Hynes (Berne, Switzerland) reported a correlation between immunohistochemical detection of c-*erb*B-2 protein and both axillary lymph node involvement and tumour nuclear grade. However, in a study of expression of the gene in primary tumours, in 79 node negative breast cancer patients the presence of the marker was not a statistically significant predictor of relapse unless combined with oestrogen receptor status. A similar study from J. Nusse (Amsterdam, The Netherlands) looked at the correlation between c-*erb*B-2 gene amplification and expression. Intense membrane staining was detected using monoclonal antibodies in 10/10 tumours with gene amplification but only 2/28 tumours with an apparently normal gene copy number. A larger study suggested that c-*erb*B-2 expression was correlated with increased tumour size and poor histological grade. An interesting observation was the demonstration of positive c-*erb*B-2 staining in 17/42 ductal carcinomas *in situ*, all being of the large cell comedo type. This may suggest an early role for over-expression of the gene in the development of a distinct histological subtype of breast cancer. R.A. Walker (Leicester, UK) reported a good correlation between c-*erb*B-2 amplification and detection of the protein product (immunochemistry) or of mRNA by *in situ* hybridisation; again the increased expression indicated poor prognosis, particularly for cases with lymph node metastases and poor differentiation of the primary carcinomas.

Alterations to other proto-oncogenes in tumours received less attention, although G. Peters (London, UK) reported studies of *int*-2, which is adjacent to the integration site for mammary tumour virus in mice. The human homologue of *int*-2 is located at 11q13 and was found to be amplified in 20/106 primary breast tumours but this did not appear to correlate with expression. Other markers in this region also showed amplification, but not as frequently, and it was suggested that another unidentified gene 5′ to *int*-2 may be important in the molecular events linked to the development of breast cancer.

The final session was devoted to possible methods of detection of individuals at increased risk through genetic predisposition. A. Howell (Manchester, UK) argued the need to identify high risk groups because general population screening by mammography is ‘a blunt tool’; the potential for over-diagnosis is enormous in that in only one of four women diagnosed with *in situ* lesions is there likely to be progression to an invasive malignancy. Several studies have found that in about 50% of women with breast cancer, multicentric and bilateral *in situ* lesions may be found. Howell argued that these may be the cases with genetic predisposition but acknowledged that this figure is an order of magnitude greater than that derived from segregation analysis of the type presented earlier by M.-C. King.

Potentially, genetic predisposition can be detected if the predisposing gene itself, or a closely linked polymorphic locus, is identified and can be used as a marker. M.-C. King described tests of genetic linkage of breast cancer to several loci in extended families with at least three living affected members. No close linkage was found to nine proto-oncogenes, most of which have been implicated in breast cancer from other studies, i.e. H-*ras* (11p15.5), K-*ras* (12p12.1), N-*ras* (1p22 or p13), c-*myc* (8q24), c-*myb* (6q22-q23), c-*erb*A2 (17q21), *int*-2 (11q13) and *raf*1 (3p25). These results strongly suggest that these oncogenes are not the sites of primary lesions for inherited breast cancer, which is consistent with the hypothesis that primary carcinogenic lesions are recessive alterations of tumour suppressor genes. Alterations in proto-oncogenes may be more closely associated with tumour progression. The results also exclude certain chromosome regions in close proximity to these markers as being the sites of predisposing genes. In particular, by using the probe for the insulin locus on 11p, in addition to the H-*ras* probe, regions of 11p associated with loss of heterozygosity in tumours were excluded. Allele loss in tumours is not always, therefore, indicative of the location of inherited predisposing genes. However, in contrast to this observation was the demonstration by MacKay that, having detected a 61% allele loss at 17p13.3 in tumours, the same probe used in linkage studies in a two generation family with seven affected members gave a suggestive positive lod score of 1.6.

The alternative strategy to identifying predisposing genes or closely linked DNA sequences for detecting individuals at risk is to identify other cellular characteristics which are strongly associated with heritable cancer-proneness. S.L. Schor (Manchester, UK; see Schor *et al.*, 1987) has detected abnormal (fetal like) migratory behaviour of skin fibroblasts in 90% premenopausal patients with familial breast cancer, in 65% of unaffected first degree relatives and in less than 10% of normal healthy controls. However, cells from 50% of patients with sporadic breast cancer also behaved in this way. Again, if we accept that less than 5% of cases are genetically predisposed this ‘marker’ may be detecting predisposition, but not heritable predisposition. Perhaps the 50% are those women with multifocal disease (see Howell, above). Similar findings were reported by N.G. Testa (Manchester, UK) who observed that in 68% (55/81) of patients with a family history of breast cancer, peripheral blood monocytes formed multinucleated cells *in vitro* (see Al-Sumidaie *et al.*, 1988). Values of 63% (15/24) were found in first degree relatives and 27% (13/47) in normal controls. However, there was no significant difference between patients with or without a family history.

Hypersensitivity to the induction of chromosome damage by ionising radiation has been observed in a number of heritable cancer-prone syndromes, notably ataxia-telangiectasia and Fanconi's anaemia (Taylor, 1983). D. Scott (Manchester, UK) reported investigations of chromosomal radiosensitivity in various phases of the cell cycle in peripheral blood lymphocytes of index cases in families with a frequency and pattern of breast cancers suggestive of heritable predisposition, in first degree relatives and in sporadic breast cancers. None of these groups showed a sensitivity greater than in normal healthy controls.

At present there is no reliable phenotypic marker of predisposition. B.H.A. Ponder (London, UK) pointed out the potential value of such a marker in identifying women who are *not* at risk. To predict high risk, the marker must indicate not only that the individual is a gene carrier but the likely expression of the gene as well.

Having accepted that there is a degree of heritable predisposition to breast cancer there is a need to provide advice to families who may be at increased risk. Ponder described his experiences in counselling such families. The essence of genetic counselling is to define the risks and to advise on appropriate action. Risks for breast cancer are difficult to define and have to be based on an averaging of risk estimates which assume that an individual *is* from a family of gene carriers and estimates based upon empirical or population data, taking into account the number of affected individuals within a family and the age of onset. Even for gene carriers the penetrance for a particular individual is unknown (although average lifetime penetrance could be high, see M.-C. King above) and may depend upon genetic factors (independently assorting modifying genes) and/or environmental influences such as parity, age at first birth and lifestyle. Having made an estimate of risk, however crude, the options presented include doing nothing in low risk cases, advising self-examination and possible changes in lifestyle, regular screening and, in the extreme, prophylactic mastectomy. Chemoprevention by e.g. tamoxifen treatment is a possibility for the future. An assessment should be made of the costs and benefits of mammography in young women with a family history of breast cancer.

The mood of the meeting was one of enthusiasm and optimism with a clear recognition of significant recent developments, particularly in the application of recombinant DNA techniques. There were predictions that linkage studies would locate predisposing genes within two years but a caution that the disease is heterogeneous and several different genes may be involved. Identification of such genes should lead to a much clearer understanding of the nature of this common disease and to improvements in detection, prevention and treatment.

### References

ALI, I.U., LIDEREAU, R., THEILLET, C. & CALLAHAN, R. (1987). Reduction to homozygosity of genes on chromosome 11p in human breast neoplasia. *Science*, **238**, 185.

AL-SUMIDAIE, A.M., LEINSTER, S.J., HART, C.A., GREEN, C.D. & McCARTHY, K. (1988). Particles with properties of retroviruses in monocytes from patients with breast cancer. *Lancet*, **ii**, 5.

BODMER, W.F., BAILEY, C.J., BODMER, J. and 10 others (1987). Localisation of the gene for familial adenomatous polyposis to chromosome 5. *Nature*, **328**, 614.

CAVENEE, W.K., DRYJA, T.P., PHILLIPS, R.A. and 6 others (1983). Expression of recessive alleles by chromosomal mechanisms in retinoblastoma. *Nature*, **305**, 779.

KNUDSON, A.G. (1971). Mutation and cancer: statistical study of retinoblastoma. *Proc. Natl Acad. Sci. USA*, **68**, 820.

KOUFOS, A., HANSEN, M.F., LAMPKIN, B.C. and 4 others (1984). Loss of alleles at loci on human chromosome 11 during genesis of Wilms' tumour. *Nature*, **309**, 170.

LUNDBERG, C., SKOOG, L., CAVENEE, W.K. & NORDENSKJULD, M. (1987). Loss of heterozygosity in human ductal breast tumours indicates a recessive mutation on chromosome 13. *Proc. Natl Acad. Sci. USA*, **84**, 2372.

NEWMAN, B., AUSTIN, M.A., LEE, M. & KING, M.-C. (1988). Inheritance of human breast cancer: evidence for autosomal dominant transmission in high-risk families. *Proc. Natl Acad. Sci. USA*, **85**, 3044.

SCHOR, S.L., SCHOR, A.M., HOWELL, A. & CROWTHER, D. (1987). Hypothesis; persistent expression of fetal phenotypic characteristics in fibroblasts is associated with an increased susceptibility to neoplastic disease. *Exp. Cell Biol.*, **55**, 11.

SEIZINGER, B.R., MARTUZA, R.L. & GUSELLA, J.F. (1986). Loss of genes on chromosome 22 in tumourigenesis of human acoustic neuroma. *Nature*, **322**, 644.

TAYLOR, A.M.R. (1983). The effect of radiation on the chromosomes of patients with an unusual cancer susceptibility. In *Radiation Induced Chromosome Damage in Man*, Ishihara, T. & Sasaki, M.S. (eds) p. 167. Liss: New York.